# Comprehensive Host Cell-Based Screening Assays for Identification of Anti-Virulence Drugs Targeting *Pseudomonas aeruginosa* and *Salmonella* Typhimurium

**DOI:** 10.3390/microorganisms8081096

**Published:** 2020-07-22

**Authors:** Julia von Ambüren, Fynn Schreiber, Julia Fischer, Sandra Winter, Edeltraud van Gumpel, Alexander Simonis, Jan Rybniker

**Affiliations:** 1 Department I of Internal Medicine, University of Cologne, 50937 Cologne, Germany; j.vonambueren@gmx.de (J.v.A.); f.schreiber@smail.uni-koeln.de (F.S.); julia.fischer@uk-koeln.de (J.F.); 2 Center for Molecular Medicine Cologne (CMMC), University of Cologne, 50931 Cologne, Germany; sandra.winter@uk-koeln.de (S.W.); edeltraud.van-gumpel@uk.koeln.de (E.v.G.); 3 German Center for Infection Research (DZIF), Partner Site Bonn-Cologne, 50937 Cologne, Germany

**Keywords:** multidrug-resistant pathogens, host-directed therapies, antibiotic drug screening, *Salmonella* Typhimurium, *Pseudomonas aeruginosa*, type 3 secretion system, antibiotic resistance

## Abstract

The prevalence of bacterial pathogens being resistant to antibiotic treatment is increasing worldwide, leading to a severe global health challenge. Simultaneously, the development and approval of new antibiotics stagnated in the past decades, leading to an urgent need for novel approaches to avoid the spread of untreatable bacterial infections in the future. We developed a highly comprehensive screening platform based on quantification of pathogen driven host-cell death to detect new anti-virulence drugs targeting *Pseudomonas aeruginosa* (*Pa*) and *Salmonella enterica* serovar Typhimurium (*S*T), both known for their emerging antibiotic resistance. By screening over 10,000 small molecules we could identify several substances showing promising effects on *Pa* and *S*T pathogenicity in our in vitro infection model. Importantly, we could detect compounds potently inhibiting bacteria induced killing of host cells and one novel comipound with impact on the function of the type 3 secretion system (T3SS) of *S*T. Thus, we provide proof of concept data of rapid and feasible medium- to high-throughput drug screening assays targeting virulence mechanisms of two major Gram-negative pathogens.

## 1. Introduction

Multidrug resistant microorganisms pose a major public health concern and are responsible for around 30,000 annual deaths and a loss of almost 1,000,000 disability-adjusted life-years in Europe alone [1]. Infections by drug-resistant Gram-negative pathogens are eminently challenging and related with an increased mortality and costs [2,3]. Development of novel antibiotic drugs targeting Gram-negative bacteria is complicated by intrinsic and acquired protective mechanisms including multidrug efflux pumps, a high mutation rate, structural properties of the cell wall and antibiotic resistance genes (e.g., antibiotic-degrading or antibiotic-inactivating enzymes like carbapenemases) determined chromosomally or acquired by horizontal gene transfer [4].

Two remarkable representatives of Gram-negative bacteria are *Pseudomonas aeruginosa* (*Pa*), a facultative pathogen that is a major cause of nosocomial infections such as pneumonia, urinary tract or bloodstream infections and *Salmonella enterica* serovar Typhimurium(*S*T), a common cause of foodborne illness which is also able to cause life threatening infections in immune compromised hosts [5,6]. Both pathogens are known for their high resistance rates [7,8,9,10]. To overcome the existing lack of new bactericidal or bacteriostatic substances, the screening for new compounds targeting virulence factors of bacteria or abrogating detrimental effects of these factors in the host seems feasible and promising.

Pathogenicity of *Pa* and *S*T is mediated by several virulence factors including lipopolysaccharide, type 4 pili and the type three secretion system (T3SS). The T3SS has already been in the focus of the development of new drugs with anti-virulence activity but no inhibitor could be implemented in a clinical usage so far [11]. For various chemical derivates, including synthetic small molecules, an inhibitory effect of the T3SS could be described. For instance, salicylidene acylhydrazide interferes with the secretion mechanisms of effector proteins by suppressing corresponding genetic signals on transcriptional levels. Also, several imidazole derivates were described to target transcription factors leading to a downregulation of virulence associated genes without impact on bacterial growth [12]. A different mode of action could be shown for 2-Imino-5-arylidenethiazolidinones derivates, which are capable of manipulating the formation of the T3SS needle complex in *S*T and thus prevent the primary infiltration of virulence factors into the host cell cytosol [13].

However, also the inhibition of other bacterial virulence factors are promising targets to fight bacterial infections: clofoctol specifically inhibits the expression of quorum sensing (QS)-controlled virulence, a bacterial cell–cell communication process, which is involved in pyocyanin production, motility and biofilm formation [14]. Furthermore, in *S*Tseveral quinazoline compounds showed a sufficient downregulation of PhoP/PhoQ-activated genes, which are crucial for environmental adaption including survival within macrophages [15,16,17]. Notably, not only bacterial factors can be targeted to abrogate pathogenesis. Manipulation of the host cell can also be used for this purpose. For example reduced intracellular bacterial growth of *S*T and *Mycobacterium tuberculosis* could be achieved by modulation of host cell kinases [18]. In *Pa* the function of the acid sphingomyelinase (ASM), an enzyme catalyzing the breakdown of sphingomyelin to ceramide and phosphorylcholine is crucial for cellular response and defense against the pathogen [19,20]. Interestingly, function of the ASM can be modulated by a large group of pharmacological compounds (also called FIASMA = functional inhibitors of acid sphingomyelinase) including several tricyclic antidepressants, calcium channel blockers and H1 antagonists [21].

Here, we established two host cell based medium-throughput screening platforms, which exploit virulence factor dependent killing of *Pa* and *S*T infected of host cells. This method provides a robust, rapid and comprehensive screening platform that theoretically allows for identification of molecules with antibiotic activity, anti-virulence and host-directed drugs, as well as antibiotic prodrugs [22]. Due to the simple batchwise setup of the assay without any required washing-steps, this method is particularly suitable for medium- and high-throughput screenings. Utilizing these platforms, we performed a proof of concept screening with 10,000 diverse chemical compounds [23]. Of note, we could identify several series of novel chemical entities that were able to protect host cells from bacteria-induced cell death without affecting the viability of the eukaryotic cells or bacterial growth, indicating an anti-virulence effect of these compounds.

## 2. Materials and Methods

### 2.1. Chemical Compounds

Gentamicin was purchased from Sigma-Aldrich (St. Louis, MO, USA) and moxifloxacin from Cayman Chemical Company (Ann Arbor, MI, USA). For the medium-throughput screening, “The world diversity set III” from Specs (Zoetermeer, Netherlands) was used.

### 2.2. Cell Culture

A549 human lung adenocarcinoma cells (American Type Culture Collection, Manassas, VA, USA) were cultured in Roswell Park Memorial Institute (RPMI)-1640 medium (ThermoFisher Scientific, Waltham, MA, USA) supplemented with 10% heat-inactivated fetal bovine serum (FBS, PAN-Biotech, Aidenbach, Germany) at 37 °C with 5% CO_2_. J774.2 mouse macrophages (Sigma-Aldrich, St. Louis, MO, USA) were grown in Dulbecco’s modified Eagle’s medium (DMEM) (ThermoFisher, Scientific, Waltham, MA, USA) supplemented with 10% FBS at 37 °C with 5% CO_2_.

### 2.3. Culture Conditions of Bacteria

*Pseudomonas aeruginosa* O1F wildtype (WT) and PAO1F∆*pscD* strains were grown in 2 x YT medium (Sigma-Aldrich, St. Louis, MO, USA). For growth inhibition assays PAO1F was grown in Mueller-Hinton broth (Sigma-Aldrich, St. Louis, MO, USA). *Salmonella* Typhimurium strains (SL1344 WT and *invA* mutant strain) were grown in brain heart infusion (BHI) medium (FisherScientific, Hampton, NH, USA).

### 2.4. Host-Cell Survival Assays

Compounds of “The world diversity set 3” from Specs (dissolved in DMSO) were pre-plated into 96-well plates at a concentration of 200 μM or 500 μM using a volume of 10 µL dH_2_O (final concentration 20 or 50 µM). As positive control, gentamicin 200 μg/mL (for *Pa*) or moxifloxacin 100 μg/mL (for *S*T) dissolved in 10 µL dH_2_O were pre-plated. As negative control DMSO (Sigma-Aldrich, St. Louis, MO, USA) in dH_2_O was added to match the DMSO concentration of the compounds (final volume 10 µL). For drug screening with *Pa* A549 cells were seeded at a density of 2 × 10^4^ cells per well in 70 µL RPMI. After pre-incubation for 3 h at 37 °C with 5% CO_2_ to ensure cell adherence, cells were infected with PAO1F with a MOI (multiplicity of infection) of 0.5 in 20 µL RPMI. After 4 h p.i. (post infection) gentamicin and moxifloxacin dissolved in 10 µL dH_2_O per well were added at a final concentration of 20 μg/mL and 10 μg/mL respectively to prevent bacterial overgrowth. After overnight incubation the fluorescent dye resazurin (Sigma-Aldrich, St. Louis, MO, USA) was added at a final concentration of 8% (v/v, 10 µL/well). Subsequently the assay plates were incubated at 37 °C with 5% CO_2_ for another 4 h. Fluorescence was measured at a wavelength of 560/590 nm (EX-max./EM-max.) using a Tecan Safire II fluorescence reader (Tecan, Maennedorf, Switzerland).

For drug screening with *S*T Specs compounds were dissolved in DSMO and then pre-plated at 10 µL each into 96-well-plates using a final drug concentration as described above (50 µM). J774.2 Mφ cells were seeded at a density of 2 × 10^4^ cells per well in 80 µL DMEM and incubated for 3 h at 37 °C with 5% CO_2_. Cells were infected with *S*T SL1344 with a MOI of 0.5 in 20 µL BHI for 3 h. To stop the infection and prevent bacterial overgrowth gentamicin (50 μg/mL) was added and the plates were incubated for another 48 h. To quantify cell viability 10 µL resazurin was added and fluorescence was measured as described above.

### 2.5. Growth Inhibition Assays

Compounds were pre-plated into 96-well plates at a concentration of 200 μM or 500 μM dissolved in 10 µL dH_2_O (final drug concentration per well: 20 or 50 µM). As controls 10 µL gentamicin (20 μg/mL), 10 µL moxifloxacin (10 μg/mL) or 10 µL DMSO were plated. Then bacteria were added at the same concentrations that were used for the host cell-based drug screening described above. Subsequently the plates were incubated overnight for *Pa* and 48 h for *S*T at 37 °C with 5% CO_2_. Finally, OD600 of each well were measured by using a Hidex Sense multimodal microplate reader (Hidex, Turku, Finland).

### 2.6. RNA-Seq in Pseudomonas Aeruginosa

For gene expression analysis *Pa* WT was grown to log-phase with G5 193 (Specs), 7-fluoroindole (7-FI) (Sigma-Aldrich, St. Louis, MO, USA) or DMSO (Sigma-Aldrich, St. Louis, MO, USA) using a final concentration of 100 µM. After RNA purification with an RNAeasy Minikit (Qiagen, Venlo, Netherlands) according to the manufacturer’s instructions, library preparation and sequencing were performed by the Cologne Center for Genomics (CCG): briefly, library preparation was performed with the TrueSeq Stranded Total RNA kit (Illumina, San Diego, CA, USA) with 1 µg total RNA input. First steps of the library preparation involved the removal of ribosomal RNA using biotinylated target-specific oligos from the RiboMinus Bacteria Kit (ThermoFisher, Scientific, Waltham, MA, USA). Following purification, the RNA was fragmented and cleaved. RNA fragments were copied into first strand cDNA using reverse transcriptase and random primers, followed by second strand cDNA synthesis using DNA Polymerase I and RNase H. These cDNA fragments then had the addition of a single “A” base and subsequent ligation of the adapter. The products were purified and enriched with PCR to create the final cDNA library. After library validation and quantification (Agilent tape station), equimolar amounts of library were pooled. The pool was quantified by using the KAPA Library Quantification Kit (VWR, Radnor, PA, USA) and the 7900HT Sequence Detection System (Applied Biosystems, Waltham, MA, USA) and sequenced on an NovaSeq6000 sequencing instrument (Illumina) and a PE100 protocol. Analysis of gene expression data were done by Rockhopper (Wellesley College, MA, USA) and Microsoft Excel (Microsoft, Redmond, WA, USA) software.

### 2.7. T3SS-Secretion Assay

Bacteria were grown overnight under T3SS inducing conditions in LB media containing 5 mM ethylene glycol-bis(2-aminoethylether) (EGTA) (Sigma-Aldrich, St. Louis, MO, USA). The next day the suspension was diluted, and compounds were added to a final concentration of 50 μM in a 50 mL tube. After another 4 h of co-incubation the supernatant was separated via centrifugation and filtered through a 0.45 μm-pore-size low protein-binding filter (ThermoFisher, Scientific, Waltham, MA, USA). Subsequently proteins were precipitated by tricholoroacetic acid (Sigma Aldrich, St. Louis, MO, USA), washed and analyzed by SDS-PAGE using Instant Blue Coomassie dye (Expedeon, Heidelberg, Germany).

### 2.8. Statistical Analysis

To prove assay-quality the non-dimensional statistical parameter Z‘-factor was used to define the data deviation of the respective controls in relation to the corresponding mean values and the dynamic range of the assay [24]. The calculated Z‘ factor (Z’ = 1 – [(3 * σ_pos_ + 3 * σ_neg_) / (µ_pos_ − µ_neg_)]) can range from 0 to 1 and is determined by the assay group’s standard deviations σ and means µ of the positive and negative control. Z‘ factor values of >0.5 indicating a reliable assay quality, which are suitable to perform high throughput screenings [24]. Statistical analysis was performed with GraphPad Prism 8.0.2 software (GraphPad, San Diego, CA, USA). The quantitative data is reported as mean value. Two-tailed Student’s t-test with confidence intervals of 95% was used for the statistical analyses of significance. *p*-values less than or equal to 0.05 were considered statistically significant.

## 3. Results

### 3.1. Assay Development and Validation for Pseudomonas Aeruginosa

**Figure 1:**
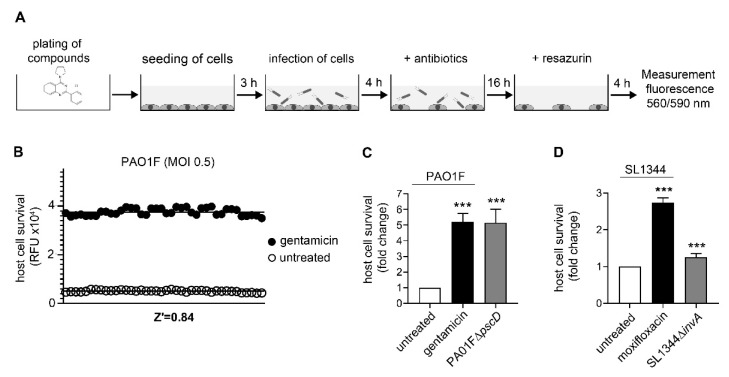
Screening assay validation for high throughput screenings and sensitivity to T3SS mediated toxicity. (**A**) Experimental setup: 96-well plates were pre-plated with compounds before A549 cells were seeded. After preincubation for 3 h to ensure cell adherence, cells were infected with *Pa* strain PAO1F at a MOI of 0.5. After 4 h p.i. antibiotics were added and the fluorescent dye resazurin was added the next day. Subsequently assay plates were incubated for another 4 h and fluorescence was measured at a wavelength of 560/590 nm. (**B**) To test for assay robustness, Z‘ factor values was calculated under various assay conditions by determining the standard deviations and means of the positive and negative controls as explained in the methods section. 2 × 10^4^ A549 cells/well were seeded into a 96 well plate and subsequently infected with the WT strain PA01F at a MOI of 0.5 in the presence of gentamicin (black dots) or left untreated (white dots). Host cell survival was determined by the fluorescent dye resazurin (RFU = relative fluorescence units). (**C**) Using an optimized assay protocol, A549 cells were co-incubated with the WT strain PA01F (white bar) and the T3SS-deficient mutant strain PA01F∆*pscD* (gray bar). As positive control gentamicin (20 μg/mL) (black bar) was added to the cells prior to infection. (**D**) For *S*T J774.2 Mφ cells were infected with a MOI of 0.5 with *S*T WT strain SL1344 (white bar) or mutant strain SL1344∆*invA* (gray bar). Moxifloxacin (10 μg/mL) were added as positive control (black bar). 4 h p.i. bacterial growth was stopped by addition of gentamicin and cells were incubated for 48 h. Subsequently resazurin was added and cell viability were measured by fluorescence reading (560/590 nm). Graphs show mean ± standard error of the mean. *** *p* < 0.001.

**Figure A1:**
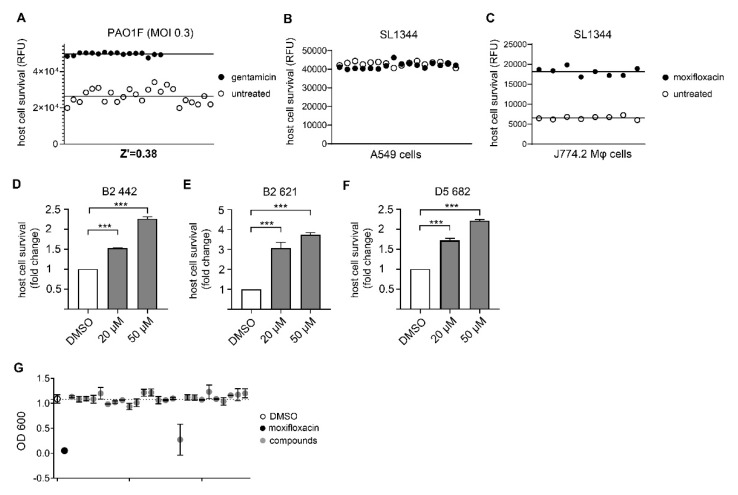
(**A**) A549 cells/well were seeded into a 96 well plate and subsequently infected with the WT strain PA01F with a MOI of 0.3 in the presence of gentamicin (black dots) or left untreated (white dots). Host cell survival was determined by addition of the fluorescent dye resazurin (RFU = relative fluorescence units). (**B**) A549 cells/well or J774.2 Mφ cells (**C**) were seeded into a 96 well plate and subsequently infected with the *S*T strain SL1344. DMSO (white dot), moxifloxacin (10 μM) (black dot). (**D**–**F**) A549 cells were infected with WT strain PA01F with a MOI of 0.5 in presence of 20 or 50 μM of different compounds (gray bars) which could be identified in the screening assay. Infection was abrogated after 4 h p.i. and cell viability was measured by fluorescence. Graphs show mean ± standard error of the mean compared to the negative control (DMSO). *** *p* < 0.001. (**G**) SL 1344 was grown for 24 h in BHI medium in presence of DMSO (white dot), moxifloxacin (10 μM) (black dot) or compounds of the Specs library (50 μM) (gray dots). Subsequently OD600 were measured using a microplate reader. Graph shows mean ± standard error of the mean.

To establish a medium to high throughput assay based on *Pa* (PA01F) dependent killing of host cells, we infected A549 cells in a batch assay that does not require washing steps (Figure 1A). Due to the frequent pulmonary infections caused of *Pa* we selected A549 cells which are commonly used as pulmonary epithelium and *Pa* infection model [25]. First, A549 cells were seeded in 96 well plates in the presence of putative anti-virulence compounds. Following infection with *Pa* for a sufficient amount of time to allow for significant host-cell damage, bacterial growth was stopped by addition of a combination of gentamicin and moxifloxacin. Both antibiotics were necessary to avoid bacterial overgrowth in all test wells. Continuous incubation overnight led to further reduction of A549 cell counts in *Pa* affected cells. To optimize assay quality, we tested several conditions including alteration of the host-cell number, incubation time, temperature or MOI. For optimal results A549 cells were seeded at a density of 2 × 10^4^ well (96 wells). After preincubation for 3 h cells were infected with a MOI of 0.5 for 4 h and antibiotics were added subsequently. After overnight incubation, resazurin was added for 4 h before cell viability was quantified by fluorescence activity at 560/590 nm (Figure 1A). We evaluated the statistical liability of our screening assays for *Pa* by calculating the Z‘factor. As representatively shown in Figure 1B, using a MOI of 0.5 resulted in a Z’ value of 0.84, while a decrease of the MOI to 0.3 led to a standard deviation and a narrow separation band of the two control groups (untreated vs gentamicin-treated cells) resulting in a decreased Z’ value of 0.38 (Figure A1A). After having determined optimal conditions, we confirmed the capability of the assay to detect disruption of *Pa* virulence by testing a T3SS-deficient strain of *Pa*. Infection of A549 cells with the mutant strain PAO1F∆*pscD*, which lacks the ability to produce pscD, an essential inner membrane T3SS component [26], led to a 5-fold increase of viability compared to cells infected with the PAO1F wildtype strain (Figure 1C). These data confirmed suitability of the assay for detection of anti-virulence drugs.

### 3.2. Assay Development and Validation for Salmonella Typhimurium

After establishment of the screening assay for *Pa* we tried to adapt the same assay for *S*T. *S*T was chosen due to comparable structural similarities and virulence factors particularly regarding the type three secretion system. Despite their supposed similarities, initial experiments failed with the same experimental conditions as used before. Importantly, no sufficient *S*T-mediated host-cell killing could be achieved in A549 cells (Figure A1B). The addition of gentamicin at an early stage prevented a sufficient cell invasion by *S*T followed by a measurable host cell cytotoxicity, whereas a prolonged incubation time led to a bacterial overgrowth of the cells, which hinder the fluorometric determination of host cell survival. To overcome this problem, we changed the in vitro model by using a macrophage cell line (J774.2 Mφ) [27]. Due to the presumable enhanced host cell invasion by *S*T, we could achieve a sufficient and timely quantifiable cell death in co-culture conditions without an imminent bacterial overgrowth.

As performed with *Pa*, we tested a large series of different conditions to achieve Z-values >0.5 (Figure A1C): In particular, we observed the necessity for a prolonged co-culture time of *S*T J774.2 Mφ to detect sufficient host-cell killing. Finally, higher gentamicin concentrations were needed to prevent bacterial overgrowth after 4 h. To determine sensitivity to anti-virulence compounds we tested a T3SS-deficient *S*T strain (SL1344∆*invA*) in our J774.2 Mφ cell-based assay. InvA is part of the inner membrane protein of the *S*T T3SS, which is genetically encoded and regulated via the *Salmonella* pathogenicity island (SPI), a key factor for *S*T virulence [28]. Thus, lack of the *invA* gene leads to an impaired function of the T3SS and a reduction of cytotoxicity in *S*T (Figure 1D). Cell viability was significantly increased using the invA deficient *S*T strain compared to the wildtype strain, indicating sufficient sensitivity of the assay for detection of potential T3SS-inhibiting or other anti-virulence compounds.

### 3.3. Identification of Novel Compounds with Anti-Virulence Activity against Pseudomonas Aeruginosa

**Figure 2:**
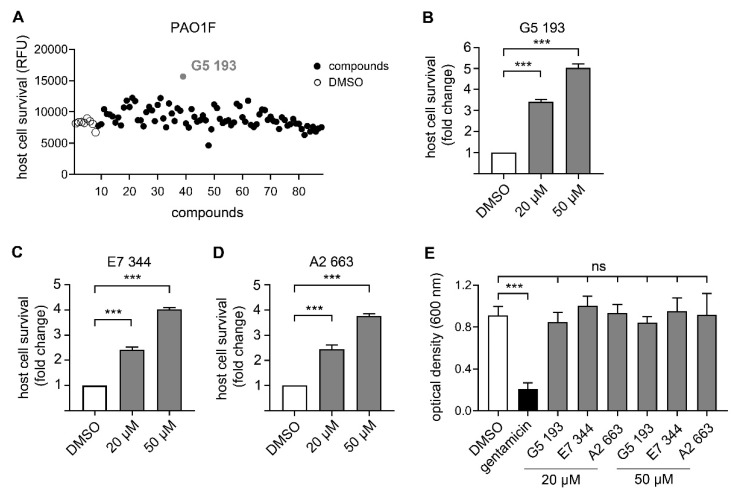
Identification of compounds with antipseudomonal activity. (**A**) A549 cells were infected with *Pa* WT strain PA01F with a MOI of 0.5 in presence of compounds of the Specs library (black dots) (20 μM). Infection was abrogated after 4 h p.i. and cell viability was measured by addition of the fluorescent dye resazurin. Graph represents one 96 well screening plate. One compound (G5 193, gray dot) shows a significant increase of cell viability compared to the negative control (DMSO; white dots). (**B**–**D**) For validation of compounds with effect on cell viability (1.5 fold increase of cell viability compared to DMSO treated cells) experiments were repeated in two different doses of 20 or 50 μM (gray bars). Host cell survival is shown as fold change compared to cells incubated with DMSO (white bars). (**E**) PA01F was grown in Miller Hinton Broth overnight in the presence of DMSO, gentamicin or compounds at a concentration of 20 or 50 μM. Subsequently the OD600 were measured by using a microplate reader. Graphs show mean ± standard error of the mean. None of the tested hit compounds had a growth inhibitory effect on *Pa* in broth. ns = non-significant; *** *p* < 0.001.

Next we performed a pilot screening with 10,000 diverse chemical small molecules using the Specs “World diversity set 3”, a library of diverse screening compounds including molecules with a molecular weight (<500 Da), bond rotation (≤10) and topological polar surface area (tPSA) (≤140Å2) [23]. Exemplary results of a screening assay in a 96-well plate format are shown in Figure 2A. A549 cells were infected with WT strain PAO1F with a MOI of 0.5 for 4 h in the presence of 80 different library compounds tested at a concentration of 20 µM. DMSO (solvent of the compounds) was used as control. Of note, for one compound (G5 193; gray dot) a remarkable increase of the RFU (relative fluorescence units) could be detected indicating increased host cell survival. Altogether, for six out of 10,000 compounds an increase of host cell survival >150% compared to the negative control could be detected. To verify our positive results, single molecules were purchased and validated by re-testing using two different concentrations (20 and of 50 μM) in the host cell survival assay which confirmed dose dependent protection of A549 cells (Figure 2B–D; Figure A1D–F) Interestingly, bacterial growth was not affected by these molecules indicating an anti-virulence effect (Figure 2E). Analysis of the chemical structures of the six substances revealed a common indoline-2-one core structure (Table 1). For compounds sharing this core structure, anti-virulence activity has been described previously: Lee et al. could show that indoline-derivates lead to a downregulation of several quorum sensing related virulence factors, which is associated with an increased host-cell survival upon infection [29].

To confirm a similar mode of action of our most potent hit (G5 193) we performed an RNA-seq experiment to analyze differential gene regulation in treated versus non-treated *Pa*. In total we were able to identify over 900 genes that were either up- or downregulated by G5 193 with at least a two-fold change. As shown for other indoline-2-one compounds we could observe a downregulation of phzA1, phzB1, phzS, pchD, pvdM and pvdS which play a relevant role in biosynthesis of the virulence factors phenazine, pyochelin and pyoverdine (Table S1). Repressing the production of these proteins at a transcriptional level can explain the host cell protective activity of the identified hit molecule. These findings are in line with the previously described effects of indoline-2-ones by Lee et al., who also could show an inhibition of quorum sensing related proteins and other virulence factors by indole and 7-hydroxyindole [29].

### 3.4. Chemical Structures of Novel Indole Compounds with Anti-Virulence Activity against Salmonella Typhimurium

**Figure 3:**
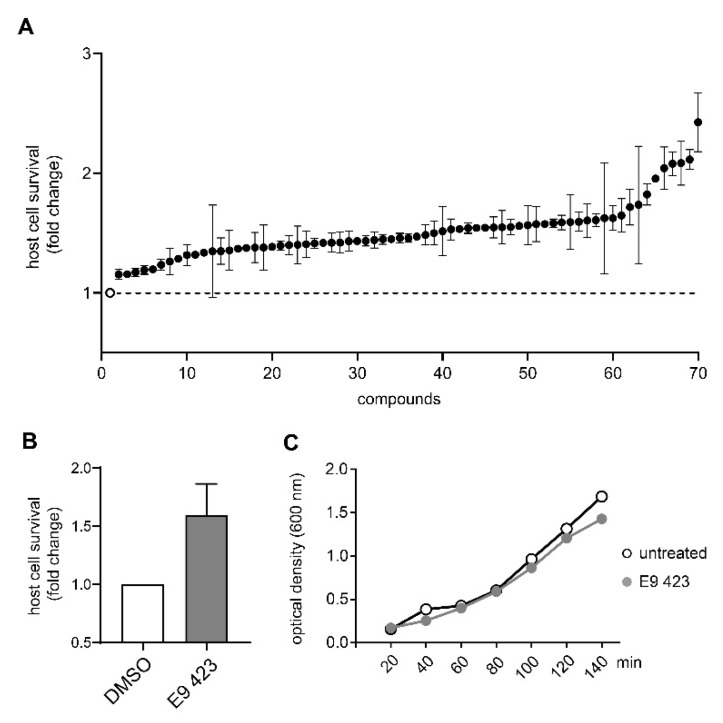
Identification of compounds with cytoprotective effect in a J774.2 Mφ cell-based screening assay targeting *S*T. (**A**) Summary of all compounds (*n* = 69) which led to an increase host cell survival in the J774.2 Mφ cell-based screening assay. Graph indicates fold change of host cell survival of infected Mφ cells treated with different compounds (black dots) compared to DMSO (white dot; dashed line) (**B**) Graph shows host cell survival of J774.2 Mφ incubated with E9 423 (gray bar) compared to cells treated with DMSO (white bar). Graphs show mean ± standard error of the mean. (**C**) Growth curve of *S*T strain SL 1344 in the presence of DMSO (white dots) or E9 423 (gray dots). Optical density measurements at 600 nm were performed at 20 min intervals.

Similar to the screening with *Pa*, we expanded our study targeting *S*T by measuring the protective effects of 10,000 synthetic small molecules (Specs “World diversity set 3”) on infected J774.2 Mφ. By screening two different species we tried to find compounds with a broad effectiveness but also to reveal specific differences between the species. After pre-plating of library compounds in 96 well plates, cells were seeded and infected with *S*T WT strain SL1344 at a MOI of 0.5 for 3 h. We could identify 69 out of 10,000 substances leading to an increase of host cell survival (Figure 3A shows a summary of the 69 substances from all screening plates). Notably, only one compound impaired bacterial growth in broth, indicating that most of the remaining hit compounds display an anti-virulence or host-cell directed effect (Figure A1G).

**Figure 4:**
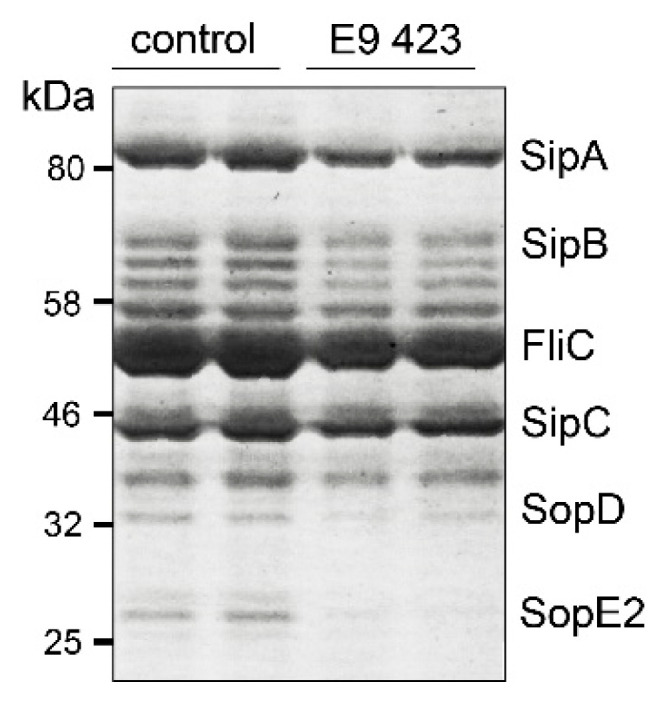
Analyzing of the T3SS-dependent secretion in presence of E9 423. Bacteria were grown overnight under T3SS-inducing conditions in LB media containing 5 mM EGTA. Subsequently bacteria were incubated with E9 423 or a control compound at a concentration of 50 μM for 4 h. After protein precipitation, proteins were washed and analyzed by SDS-Page using a Coomassie staining. Representative example of two individual experiments performed in duplicates.

To get a better understanding of the mechanisms of action of the compounds, we performed an in depth structural analysis. In line with the results of the *Pa* screening, we also could identify compounds with an indoline-2-one core structure, which are known to have a anti-virulence capability in several bacterial species [29]. The unique chemical structure of E9 423 (Table 2) awakened our interest. This compound showed a cytoprotective effect with an increase of host cell survival to 159% compared to cells treated with DMSO (Figure 3B). Furthermore, bacterial growth was not affected by E9 423 (Figure 3C). Thus, we hypothesized a possible T3SS-inhibitory effect of E9 423. To test this, we incubated bacteria overnight in T3SS inducing conditions by adding EGTA into LB broth. Afterwards bacteria were incubated with compounds for 4 h. After protein precipitation, the proteins of the supernatant were washed and analyzed by SDS-PAGE. Remarkably, bacteria treated with E9 423 showed decreased secretion of T3SS proteins in the supernatant (Figure 4). These data indicate that E9 423 protects infected Mφ by a T3SS-inhibitory effect.

## 4. Discussion

In this study, we describe the development of a highly comprehensive screening platform for detection of new anti-virulence drugs targeting *Pseudomonas aeruginosa* and *Salmonella* Typhimurium. Due to its simple batchwise setup without any required washing-steps this platform is in particular suitable for medium- and high-throughput screenings.

Functionality was first confirmed with T3SS-deficient bacterial strains, which indicated the ability to detect substances with anti-virulence activity. By testing 10,000 compounds of the Specs “World diversity set 3” we were able to identify several novel compounds with cytoprotective effects. Importantly, one novel inhibitor of the *S*T T3SS could be identified. Several other hit substances with chemical structures of unknown function provide the possibility for further research and the potential foundation of development of new drugs.

Since their implementation decades ago, usage of antibiotics has been accompanied by appearance of drug-resistant strains. While in the past development of new bactericidal and bacteriostatic substance was in the focus of interest, nowadays substances targeting virulence factors of bacteria and host-directed therapeutics attract more attention, not least due to the lack of new conventional antibiotics [11,30,31]. The difficulty in identification of truly novel antibiotics due to intrinsic resistance is also reflected in our screening results of 10,000 highly diverse compounds: Only one hit compound showed a reduction in bacterial viability in broth which was comparable to the conventional antibiotic moxifloxacin (Figure A1G). Nonetheless, exploiting this chemical library, several compounds with cytoprotective effects could be identified.

In both infection models, the treatment with various indoline-2-one derivates was associated with an increased host cell survival. As previously described we could not detect any growth-inhibitory effect of indoline-2-ones on *Pa* or *S*T. [32]. However, indole and indoline derivates such as 7-fluoroindole are known to inhibit a series of virulence factors of *Pa* such as quorum sensing, swarming and synthesis of pyocyanin, pyochelin and pyoverdine [29]. In *S*T an inhibition of motility as well as a reduced expression of SPI-1 encoded virulence genes in response to indoline exposure were described previously [33]. Indole was also shown to reduce flagellar motility and in vitro invasion of *S*T [34]. Furthermore Lee et al. described various impacts of indole and 7-hydroxyindole on the regulation of *Pa* virulence factors by microarray experiments [29]. By exploiting RNA-seq, we used a similar approach and were able to show indoline-2-one mediated downregulation of phzA1, phzB1, phzS, pchD, pvdM and pvdS, genes which are crucial for the biosynthesis of phenazine, pyochelin and pyoverdine [33,34]. The substance also led to a substantial downregulation of the T3SS export protein pscI. Inhibition of these factors may explain the host cell protective activity of our identified hit molecules.

Interestingly, differences in the efficacies of structurally distinct indoline-2-one derivates in *Pa* and *S*T could be observed: Only one compound (E7 344) was associated with an increased host cell survival of > 150% for both pathogens. This observation indicates that besides the species-overarching effects of indoline-2-one derivates, also some derivates are species-specific most likely due to differences in transcriptional regulation of virulence factors.

In our study we also identified one novel compound (E9 423), which was able to reduce T3SS-mediated exotoxin secretion in *S*T. This compound is not an indoline-2-one derivate and, to our knowledge, similar chemical structures have not been described in the context of anti-bacterial or anti-T3SS activity. Further analyses are needed clarify the exact mechanism of E9 423 in *S*T.

Interestingly, we identified several cytoprotective compounds which failed to inhibit the T3SS of *S*T. An in-depth structural analysis revealed striking similarities in some of these compounds with known inhibitors of NLPR3 (NOD-, LRR- and pyrin domain-containing protein 3), a key regulator of pyroptotic cell death (Table A1) [35,36]. This indicates that these substances rather target host cell functions and that the observed cytoprotective effect may be mediated by preventing regulated necrotic cell death such as pyroptosis. Pyroptosis occurs upon activation of the innate immune response and the associated release of pro-inflammatory cytokines resulting in cell death in response to *S*T [37] These findings indicate that the here described host cell-based drug screening assays can identify inhibitors targeting both bacterial virulence factors as well as their effector mechanisms on the host side.

In summary, this study provides a robust and cost effective screening platform suitable for medium- and high-throughput screens that enables to identify not only molecules with antibiotic activity, but also anti-virulence and host-directed drugs, such as antibiotic prodrugs. By screening of 10,000 chemical compounds we could detect several substances with anti-virulence properties. In particular, one novel *S*T T3SS inhibitor and several novel indole-like inhibitors for one of which we identified dysregulation of *Pa* virulence associated genes by RNAseq. Finally, three potential inhibitors of necrotic host cell death could be identified in our *S*T screening. Further studies are now required to clarify the exact mechanism of action of these compounds. In addition, larger screening campaigns with more compounds should now be performed for the identification of novel inhibitors targeting these important Gram-negative pathogens.

## Figures and Tables

**Table 1:** Chemical structures of the six substances with antipseudomonal activity.

Internal Number	Specs-ID	Chemical Structure
G5 193	AJ-292/43278258	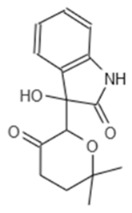
E7 344	AK-968/11036034	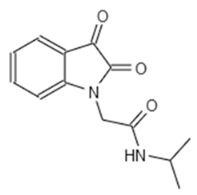
A2 663	AQ-911/40696225	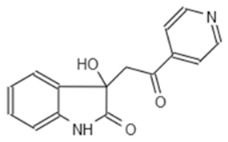
B2 442	AK-918/42028178	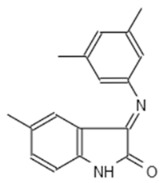
B2 621	AQ-900/41921933	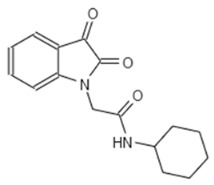
D5 682	AG-219/3696225	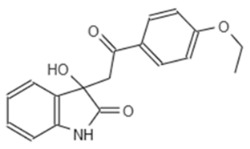

**Table 2:** Chemical structure of E9 423.

Internal Number	Specs-ID	Chemical Structure
E9 423	AN-584/43416482	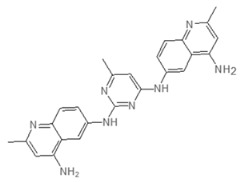

## Supplementary Materials

The following are available online at https://www.mdpi.com/2076-2607/8/8/1096/s1, Table S1: RNAseq *Pa*.

## Appendix A

**Table A1:** Selection of compounds with similarity to known NLRP3 inhibitors and cytoprotective effects in our *Salmonella*-infection model.

Internal Number	Specs-ID	Chemical Structure
D1 666	AQ-750/42209760	
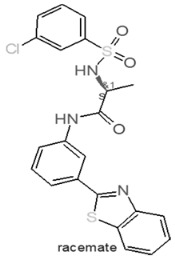
A2 668	AF-399/42309870	
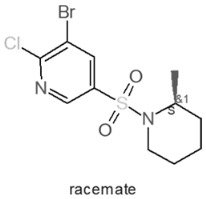
G6 120	AO-079/15259251	
