# Supplementary figures and images for: Comprehensive Host Cell-Based Screening Assays for Identification of Anti-Virulence Drugs Targeting Pseudomonas aeruginosa and Salmonella Typhimurium
Source: Microorganisms. 2020 Jul 22;8(8):1096. doi: 10.3390/microorganisms8081096 (PMC7463580; doi:10.3390/microorganisms8081096)

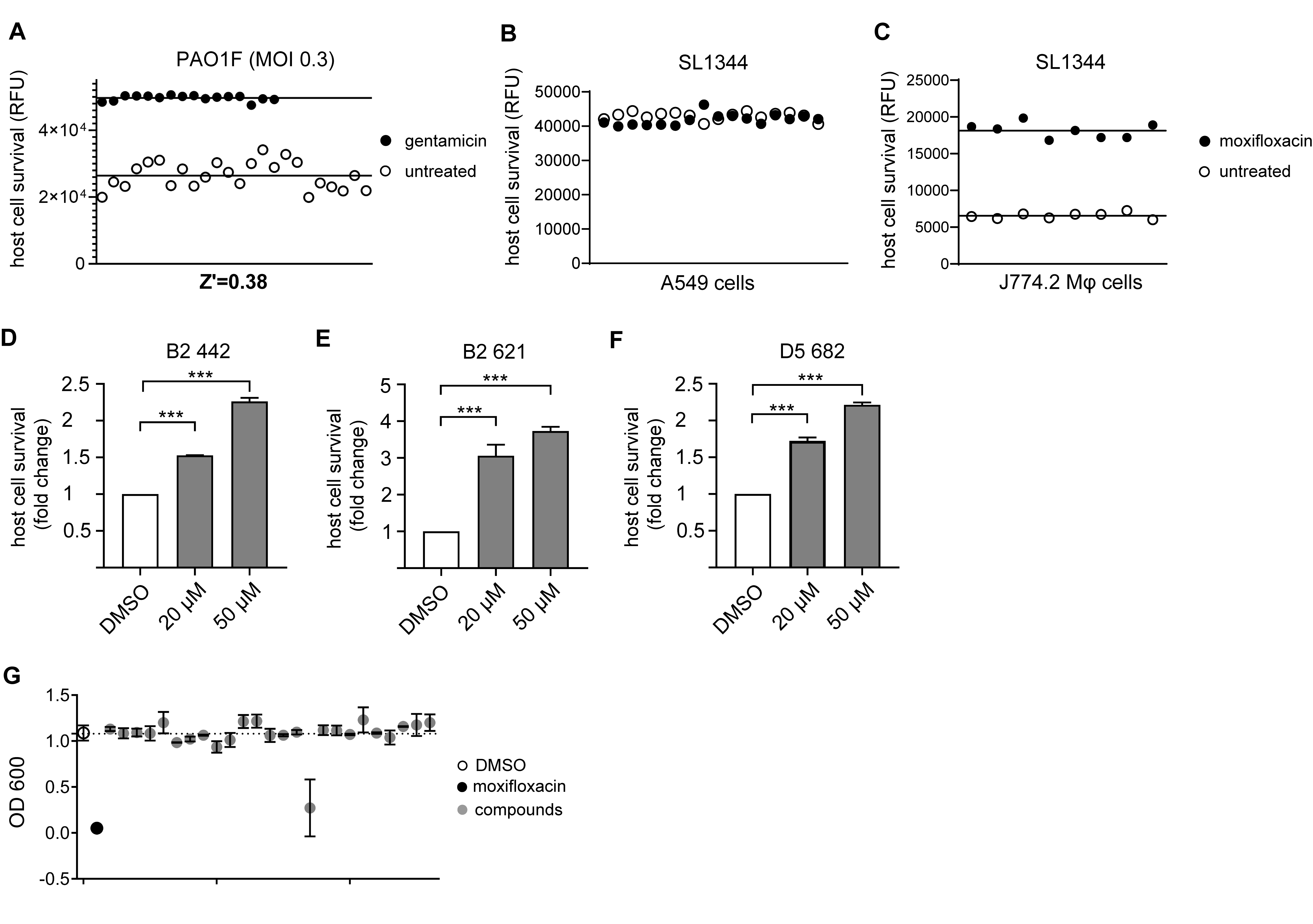

Supplement: Supplementary file 1 [file microorganisms-08-01096-s001.zip › microorganisms-876474-supplementary.tif]
